# Checklist of the Tribe Eucosmini Obraztsov, 1946 (Lepidoptera: Tortricidae: Olethreutinae) from Taiwan

**DOI:** 10.3390/insects16080819

**Published:** 2025-08-07

**Authors:** Yinghui Sun, John W. Brown, Ming Liu, Qiangcheng Zeng, Houhun Li

**Affiliations:** 1 College of Life Sciences, Dezhou University, Dezhou 253023, China; sunyh8789@dzu.edu.cn (Y.S.);; 2 College of Life Sciences, Nankai University, Tianjin 300071, China; 3 National Museum of Natural History, Washington, DC 20013-7012, USA; tortricidae.jwb@gmail.com; 4 College of Life and Geographic Sciences, Kashi University, Kashi 844000, China; 5 Xinjiang Key Laboratory of Biological Resources and Ecology of Pamirs Plateau, Kashi 844000, China

**Keywords:** Olethreutinae, description, host plants, Taiwan province, taxonomy, tortricid fauna

## Abstract

This study presents an updated and detailed inventory of the tortricid tribe Eucosmini found in Taiwan Province, China, highlighting 26 genera and 53 species. Several taxonomic novelties were revealed: The genus *Coenobiodes* is newly recorded for Taiwan Province, *Hermenias semicurva* is newly reported for China, and six species are newly recorded for Taiwan. Most Eucosmini species in Taiwan are widespread in eastern Asia and/or the Palearctic, but 12 (23%) are endemic to Taiwan. Biogeographical distributions are provided for each species, and a list of the specimens examined is provided where applicable. These findings underscore Taiwan’s status as a biodiversity hotspot and offer crucial data for understanding regional and global biodiversity patterns.

## Simple Summary

We provide a list of 53 species in 26 genera of the tortricid tribe Eucosmini from Taiwan, China. We examined 694 specimens collected from more than 60 sites between 1921 and 2005. The specimens examined are deposited in the National Museum of Natural History, Washington, DC, USA (USNM), and the McGuire Center for Lepidoptera, Florida Museum of Natural History, Gainesville, Florida, USA (MGCL). This study is complementary to previous studies on the Taiwan tortricids and contributes to a comprehensive survey of Chinese Lepidoptera. It also provides basic data for systematic and biogeographic research on the world’s leaf-roller moths.

## 1. Introduction

Tortricidae is the most species-rich family of the so-called microlepidoptera, with over 11,500 described species distributed worldwide [1]. The family includes some of the most economically important pests of fruit on the planet [2], and hence, is of considerable quarantine significance. The family comprises three subfamilies (i.e., Chlidanotinae, Olethreutinae, and Tortricinae) and 17 tribes, the monophyly of which has been demonstrated through molecular phylogenetic analyses [3,4].

The Tortricidae of Taiwan have received considerable attention [5,6], with Kawabe et al. listing 223 species. Razowski [7] added 12 more, bringing the total to 235, and most recently, Sun and co-authors [8,9,10,11,12] have described several new species and added new records to the known fauna.

Erected by Obraztsov in 1946 [13], the tortricid tribe Eucosmini (Olethreutinae) encompasses approximately 120 described genera and over 1500 species globally [1]. The tribe is present in every biogeographic region but appears to be most diverse in the Holarctic. Despite the fact that the tribe includes numerous agricultural and forestry pests, it remains understudied in East Asia. Prior to this investigation, 46 species distributed across 25 genera were known from Taiwan [5,6]. The purpose of this contribution is to update the knowledge of the tribe in Taiwan and present records of species new to the fauna, creating a new baseline for future investigations. The information presented herein will provide a more robust foundation for the conservation of tortricid biodiversity and contribute to the development of more effective management strategies for economically relevant tortricid species.

## 2. Materials and Methods

### 2.1. Study Area

Taiwan Province is an island in the northwestern Pacific Ocean between the East and South China Seas, located at the eastern edge of the Eurasian tectonic plate. It covers an area of approximately 35,808 km^2^, with high mountains in the eastern two-thirds, consisting primarily of five ranges extending parallel to the east coast, and low to rolling plains in the western one-third. Its position at the intersection of the tropical and subtropical zones creates a hot, wet climate throughout the year.

### 2.2. Collecting Methods and Specimens Examined

Adult moths were collected mainly at ultraviolet (UV) light and in UV bucket traps during multiple expeditions to Taiwan, primarily by Donald and Mignon Davis and John Heppner and H. Wang (ca. 1980–1990). However, a considerable number of specimens also were collected by Issiki in 1920–1930, Shibata in the 1970s, and Kawabe in the 1980s.

### 2.3. Depositories

The specimens examined in this study are systematically curated in two institutions: The National Museum of Natural History, Washington, DC, USA (USNM); and the McGuire Center for Lepidoptera, Florida Museum of Natural History, Gainesville, Florida, USA (MGCL). In addition to the examination of specimens, we scoured the published literature for records of Eucosmini from Taiwan.

### 2.4. Institutional Abbreviations

Institutional abbreviations employed in this study are defined for cross-reference clarity

AMNH—American Museum of Natural History, New York, USA

ANIC—Australian National Insect Collection, CSIRO Division of Entomology, Canberra, Australia

CMNH—Carnegie Museum of Natural History, Pittsburgh, Pennsylvania, USA

EIHU—Entomological Institute, Hokkaido University, Sapporo, Japan

MGCL—McGuire Center for Lepidoptera, Florida Museum of Natural History, Gainesville, Florida, USA

MCZ—Museum of Comparative Zoology, Harvard University, Cambridge, Massachusetts, USA

MGAB—Muzeul de Istorie Naturala Grigore Antipa, Bucharest, Romania

MNHN—Museum National d’Histoire Naturelle, Paris, France

NHMB—Naturhistorisches Museum, Basel, Switzerland

NHMUK—The Natural History Museum, London, United Kingdom [=formerly British Museum (Natural History)]

NMNS—National Museum of Natural Science, Taichung, Taiwan

OPU—Entomological Laboratory, Osaka Prefecture University (formerly University of Osaka Prefecture), Sakai, Japan

OUM—Hope Museum, Oxford University Museum of Natural History, Oxford, United Kingdom

TD—Type depository

TL—Type locality

USNM—National Museum of Natural History, Washington, DC, USA

ZIS—Zoological Institute, Russian Academy of Sciences, St. Petersburg (Leningrad), Russia (also ZMAS, ZIN, RAS)

## 3. Results

We document 53 species across 26 genera, including several taxonomic novelties. Notably, the genus *Coenobiodes* Kuznetzov, 1973, is newly recorded for Taiwan Province, while *Hermenias semicurva* (Meyrick, 1912) is newly reported for China. Additionally, six species are recorded from Taiwan for the first time: *Coenobiodes acceptana* Kuznetzov, 1973, *Epiblema autolitha* (Meyrick, 1931), *Epinotia majorana* (Caradja, 1916), *Rhopobota antrifera* (Meyrick, 1935), *Spilonota albicana* (Motschulsky, 1866), and *Spilonota lechriaspis* (Meyrick, 1932).

Most Eucosmini species recorded in Taiwan are fairly widespread throughout eastern Asia and/or the Palearctic region. However, 12 species (23%) appear to be endemic to Taiwan—*Epiblema alishana*, *Epinotia fujisawai*, *Epinotia melanosticta*, *Epinotia monticola*, *Eucosma pentagonaspis*, *Heleanna melanomochla*, *Hermenias pilushina*, *Lepteucosma blandoides*, *Lepteucosma dasyueshanensis*, *Notocelia donaldana*, *Notocelia kurosawai*, and *Zeiraphera taiwana*. The genera *Epinotia*, *Lepteucosma*, and *Rhopobota* are represented by the most species, with nine, four, and four, respectively. Additionally, two species—*Crocidosema lantana* and *C. plebejana*—are nearly cosmopolitan, distributed in many places around the globe.

No specimens of the following taxa attributed to the fauna of Taiwan were found during our investigations: *Epinotia salicicolana* Kuznetzov, 1968; *Epinotia piceicola* Kuznetzov, 1970; *Eucosma pentagonaspis* Meyrick, 1931; *Hermenias pilushina* Razowski, 2000; *Kennelia albifacies* (Walsingham, 1900); *Lepteucosma blandoides* Lu *et* Hsu, 2024; *Lepteucosma ceriodes* Meyrick, 1909; *Lepteucosma dasyueshanensis* Lu *et* Hsu, 2024; *Peridaedala japonica* Oku, 1979; and *Rhopalovalva catharotorna* (Meyrick, 1935). The absence of these species in our survey material underscores the fact that considerable effort remains before an inventory of the Eucosmini of Taiwan can be considered complete and comprehensive.

Below we present an annotated list of the Eucosmini, along with brief synonymies, material examined, host plants (when known), and geographic distribution.

### 3.1. Assulella *Kuznetzov, 1973*

*Assulella* Kuznetzov, 1973: 691. Type species: *Eucosma litigiosa* Meyrick, 1912.

#### *Assulella litigiosa* (Meyrick, 1912)

*Eucosma litigiosa* Meyrick, 1912: 867. TL: India, Assam, Khasi Hills. Holotype (male): NHMUK.

*Eucosma litigosa* Diakonoff, 1959: 55. [misspelling of *litigiosa*].

*Assulella kuznetzovi* Diakonoff, 1983: 23. TL: China, Li-Kilang. Holotype: MGAB, male.

*Assulella lithocosma* Diakonoff, 1983: 25. TL: Indonesia, Sumatra, Mt. Bandahara, Bivouac Four. Holotype: RMNH, male.

*Peridaedala litigiosa* (Meyrick): Kawabe, 1992: 108.

**Material examined.** 1 ♂, Chiai Hsien, Alishan, 2270 m, 8-10-VIII-1985, A. Kawabe; 2 ♂♂, 5 ♀♀, Chiai Hsien, Alishan, 2270 m, 8-10-VIII-1985, A. Kawabe; 1 ♀, Chiai Hsien, Alishan, 2270 m, 3-I-1986, H. Nakajima; 2 ♀♀, Hualien Hsien, Hohuanshan, 3100 m, 30-VII-1-VIII-1983, A. Kawabe; 1 ♂, Nantou Co., Mei-feng, 30 km S Tayuling, 2200 m, 1-8-VI-1980, D. R. Davis; 1 ♂, Taiwan: Tattaka, 19-VII-1925, S. Issiki; 1 ♀, Taiwan, S. Issiki. [USNM]. 1 ♀, Nantou Co., Meifeng, 2100 m, 6-9-IX-1988, J. Heppner; 4 ♂♂, Taichung Co., Chingshan, 1100 m, 31-VIII/4-IX-1988, J. Heppner & H. Wang; 3 ♂♂, Taichung Co., Chingshan, 2750 m, 25-X-1984, J. Heppner & H. Wang; 1 ♂, 2 ♀♀, Hsinchu Co., Kuangwu For. Sta., 2000 m, 18-25-VIII-1988, J. Heppner & H. Wang; 1 ♀, Ilan Co., Taipingshan, 1900 m, 13-V-1989, J. Heppner & H. Wang; 1 ♂, 3 ♀♀, Taichung Co., Chingshan, 1100 m, 31-VIII/4-IX-1988, J. Heppner & H. Wang; 1 ♀, Taichung Co., Anmah-shan, 2250 m, 29-30-VIII-1988, J. Heppner & H. Wang. [MGCL].

**Distribution.** China (Guangxi, Taiwan, Yunnan), India and Indonesia.

### 3.2. Coenobiodes *Kuznetzov, 1973*

*Coenobiodes* Kuznetzov, 1973: 687. Type species: *Coenobiodes acceptana* Kuznetzov, 1973.

**Remarks.** No species in this genus had been recorded in Taiwan prior to this study.

#### *Coenobiodes acceptana* Kuznetzov, 1973

*Coenobiodes acceptana* Kuznetzov, 1973: 689. TL: Japan, Honshu, Rokksan, near Kobe. Holotype (male): MGAB.

**Material examined.** 1 ♂, Kaohsiung Co., Liukuei, Shanpin For., 750 m, 16-23-III-1990, J. Heppner & H. Wang. [MGCL]. (Adult Number: TORT12086 ♂, Genitalia Slide Number: YH-Sun FL190 ♂)

**Distribution.** China (Anhui, Guizhou, Taiwan, Zhejiang) and Japan.

**Remarks.** This species is recorded from Taiwan for the first time.

### 3.3. Crocidosema *Zeller, 1847*

*Crocidosema* Zeller, 1847: 721. Type species: *Crocidosema plebejana* Zeller, 1847.

*Crocidosoma* Walker, 1863: 279. [misspelling of *Crocidosema*].

*Heligmocera* Walsingham, 1892: 507. Type species: *Heligmocera calvifrons* Walsingham, 1891.

*Parasuleima* Clarke, 1965: 77. Type species: *Crocidosema insulana* Aurivillius, 1922.

#### 3.3.1. *Crocidosema lantana* (Busck, 1910)

*Crocidosema lantana* Busck, 1910: 132. TL: Hawaiian Islands, Oahu, Tantalus. Holotype: USNM, male.

*Epinotia lantana* (Busck): Heinrich, 1923: 190.

*Eucosma polyphaea* Turner, 1926: 130.

*Eucosma eridela* Turner, 1946: 202.

*Eucosma tornocosma* Turner, 1946: 205.

*Eucosma apicinota* Turner, 1946: 206.

*Eucosma perversa* Turner, 1946: 207.

*Eucosma phaedropa* Turner, 1946: 209.

**Material examined.** 1 ♂, Taitung Hsien, Chihpen spa, 200 m, 28-III-1984, A. Kawabe. [USNM].

**Host plants.** Anonaceae: *Annona muricata*; Fabaceae: *Parkinsonia aculeata* L.; Lamiaceae: *Mentha* sp.; Verbenaceae: *Lantana camara* Linn.

**Distribution.** China (Hainan, Taiwan, Yunnan), Australia, Mexico, Sri Lanka and the Americas.

**Remarks.** This species has been introduced to many places around the world for the control of weedy *Lantana*.

#### 3.3.2. *Crocidosema plebejana* Zeller, 1847

*Crocidosema plebejana* Zeller, 1847: 721. TL: Sicily, Syracuse. Syntype (unknown sex): Unknown.

*Penthina altheana* Mann, 1855: 555. TL: Germany. Syntype(s) (unknown sex): Unknown.

*Paedisca lavaterana* Millière, 1863: 34. TL: France, Marseille. Syntype(s) (unknown sex): MNHN.

*Grapholitha peregrinana* Möschler, 1866: 139. TL: France, Nice. Syntype(s) (unknown sex): Unknown.

*Steganoptycha obscura* Wollaston, 1879: 341.

*Crocidosema plebeiana* Meyrick, 1881: 659. [misspelling of *plebejana*].

*Proteopteryx blackburnii* Butler, 1881: 393. TL: Hawaiian Islands, Maui. Syntype(s) (unknown sex): NHMUK.

*Grapholitha excitana* Möschler, 1890: 333. TL: Puerto Rico. Syntype(s) (unknown sex): Unknown.

*Crocidosema ptiladelpha* Meyrick, 1917: 18. TL: Peru, Lima. Lectotype (male): NHMUK.

*Crocidosema insulana* Aurivillius, 1922: 267.

*Crocidosema synneurota* Meyrick, 1926: 276.

*Eucosma charmera* Turner, 1946: 202. TL: Australia, New South Wales, Ebor. Lectotype (male): ANIC.

*Eucosma tornocycla* Turner, 1946: 210. TL: Australia, Queensland, Eungella. Lectotype (female): ANIC.

*Crocidosema iris* Diakonoff, 1984: 389. TL: Indonesia, West Sumba, Rua. Holotype (male): NHMB.

*Crocidosema bostrychodes* Diakonoff, 1992: 46. TL: Madagascar, Sambiano, Nosy Be. Holotype (male): MNHN.

**Material examined.** 1 ♂, 1 ♀, Pintung Hsien, Kenting Park, 100 m, 26-27-III-1984, A. Kawabe; 1 ♂, Taihoku, 28-X-1923, S. Issiki. [USNM].

1 ♂, Pingtung Co., Kenting Park, 255 m, 23-28-IV-1989, J. Heppner & H. Wang; 1 ♀, Pingtung Co., Kenting Park, 50 m, 1-5-IX-1983, J. Heppner; 1 ♀, Pingtung Co., Kenting Park, 255 m, 9-15-III-1990, J. Heppner & H. Wang; 1 ♂, 1 ♀, Nantou Co., Tungpu, 1150 m, 1-VII-1985, J. Heppner & H. Wang. [MGCL].

**Host plants.** Apiaceae: *Foeniculum piperatum* A. P. de Candolle; Cucurbitaceae: *Cucurbita pepo* L.; Malvaceae: *Abelmoschus esculentus* (L.) Moench, *Abutilon avicennae* Gaertn., *A*. *indicum* (Torner) Sweet, *Althaea rosea* (L.) Cav., *Anoda cristata* (L.) Slecht, *Gossypium* sp., *Lavatera arborea* L., *Hibiscus militaris* Cav., *H*. *rosa-sinensis* L., *Kosteletzkya althaeifolia* (Champ.) A. Gray, *Malva parviflora* L., *Malvastrum* sp., *Malvaviscus* sp., *Sida rhombifolia* L., *S*. *cordifolia* L.; Myrtaceae: *Eucalyptus* sp.; Rosaceae: *Crataegus* sp.; Salicaceae: *Salix safsaf* Forsk.; Turneraceae: *Turnera ulmifolia* L.

**Distribution.** China (Taiwan), the Americas, Australia, Europe, India, Iran, Japan and North Africa. This species is a common pest of cotton in many places.

**Remarks.** Recent molecular studies indicate that *C. plebejana*, as currently circumscribed, may represent up to four different cryptic species [14].

### 3.4. Emrahia *Kocak, 1981*

*Emrahia* Kocak, 1981: 120. [replacement name for *Scoliographa*].

*Scoliographa* Diakonoff, 1975: 312. Type species: *Argyroploce hoplista* Meyrick, 1927. [preoccupied].

#### *Emrahia hoplista* (Meyrick, 1927)

*Argyroploce hoplista* Meyrick, 1927: 340. TL: Indonesia, Sumatra, Sinabaeng. Lectotype (male): NHMUK.

*Scoliographa hoplista* (Meyrick): Kawabe, 1992: 108.

*Emrahia hoplista* (Meyrick): Brown, 2005: 271.

**Material examined.** 1 ♂, Taihoku, VI-1921, S. Issiki; 1 ♀, Taihoku, 5-X-1933, S. Issiki; 1 ♀, Taihoku, 6-X-1933, S. Issiki; 1 ♂, Taiwan: Tipononsen, E-X-1936, S. Issiki; 1 ♀, Taiwan: Taihoku, 23-VI-1935, S. Issiki. [USNM].

**Host plants.** Acanthaceae: *Barleria* sp.; Lamiaceae: *Perilla frutescens* (L.) Britton.

**Distribution.** China (Taiwan), Indonesia, Thailand, Japan and Sumatra.

### 3.5. Epiblema *Hübner, [1825]1816*

*Epiblema* Hübner, [1825]1816: 375. Type species: *Phalaena (Tinea) foenella* Linnaeus, 1758.

*Epiblemma* Hübner, [1825]1816: 63. [misspelling of *Epiblema*].

*Cacochroea* Lederer, 1859: 331. Type species: *Paedisca grandaevana* Lienig et Zeller, 1846.

*Monosphragis* Clemens, 1860: 354. Type species: *Monosphragis otiosana* Clemens, 1860.

*Euryptychia* Clemens, 1865: 140. Type species: *Euryptychia saligneana* Clemens, 1865.

*Eurytychia* Heinrich, 1923: 137. [misspelling of *Euryptychia*].

#### 3.5.1. *Epiblema alishana* Kawabe, 1986

*Epiblema alishana* Kawabe, 1986: 82. TL: Taiwan, Chiai Hsien, Alishan. Holotype (male): USNM.

**Material examined.** Holotype: ♂, (Alishan) Chiai Hsien, Formosa, 7-VIII-1971, Y. Shibata. Paratype: 1 ♂, (Alishan) Chiai Hsien, Formosa, 2300 m, 8-VIII-1974, Y. Shibata. [USNM].

**Distribution.** China (Taiwan).

#### 3.5.2. *Epiblema autolitha* (Meyrick, 1931)

*Eucosma autolitha* Meyrick, 1931: 145. TL: Japan, Honshu, Hashimoto. Holotype (male): NHMUK.

*Epiblema autolitha* (Meyrick): Nasu, 1980: 33.

*Notocelia autolitha* (Meyrick): Kawabe, 1982: 133.

**Material examined.** 1 ♀, Taichung Co., Chingshan, 1100 m, 31-VIII/4-IX-1988, J. Heppner & H. Wang. [MGCL]. (Adult Number: TORT12074 ♀, Genitalia Slide Number: YH-Sun FL181 ♀)

**Host plant.** Lauraceae: *Tachilus thunbergii* Sieb. *et* Zucc.

**Distribution.** China (Anhui, Beijing, Fujian, Gansu, Guangdong, Guizhou, Hebei, Heilongjiang, Henan, Hubei, Hunan, Jilin, Shaanxi, Sichuan, Taiwan, Tianjin, Zhejiang), Japan and Korea.

**Remarks.** This species is recorded for the first time in Taiwan.

#### 3.5.3. *Epiblema foenella* (Linnaeus, 1758)

*Epiblema foenella* (Linnaeus): Kennel, 1921: 583.

*Phalaena (Tinea) foenella* Linnaeus, 1758: 536. TL: Europe. Syntype(s) (unknown sex): Unknown.

*Epiblema foenella ab. accentana* Caradja, 1916: 67. TL: Russia, Khabarovsky Krai, Radd. Lectotype (male): MGAB.

*Epiblema foenella form acclivella* Uffeln, 1912: 136. TL: Europe. Syntype(s) (unknown sex): Unknown.

*Epiblema foenella ab. albrechtella* Meyer, 1911: 95. TL: Europe. Syntype(s) (unknown sex): Unknown.

*Epiblema foenella ab. circumflexana* Caradja, 1916: 67. TL: Russia, Khabarovsky Krai, Radd. Lectotype (male): MGAB.

*Grapholita clavigerana* Walker, 1863: 389. TL: China, Shanghai. Holotype (male): NHMUK.

*Epiblema foenella form effusana* Uffeln, 1912: 136. TL: Russia, Amur. Syntype(s): (unknown sex): Unknown.

*Ephippiphora faeneana* Guenée, 1845: 176. [misspelling of *foenella*].

*Epiblema focnella* Escherich, 1931: 341. [misspelling of *foenella*].

*Tortrix foenana* Haworth, 1811[1812]: 439. [misspelling of *foenella*].

*Paedisca foeneana* Treitschke, 1830: 106. [misspelling of *foenella*].

*Phalaena fonella* Zeller, 1853: 208. [misspelling of *foenella*].

*Epiblema foenella form fracta* Popescu-Gorj, 1965: 166. TL: Romania. Holotype (male): MGAB.

*Phalaena hochenwartiana* Scopoli, 1772: 117. TL: Europe. Syntype(s) (unknown sex): Unknown.

*Pyralis pflugiana* Fabricius, 1787: 227. TL: Denmark. Syntype(s) (unknown sex): Unknown.

*Tortrix scopoliana* Denis *et* Schiffermüller, 1775: 129. TL: Austria. Syntype(s) (unknown sex): Unknown.

*Epiblema foenella* ab. *separana* Krulikowsky, 1908: 18. TL: Russia. Syntype(s) (unknown sex): Unknown.

*Sciaphila sinicana* Walker, 1863: 347. TL: China, Shanghai. Holotype (male): NHMUK.

*Phalaena (Tortrix) tibialana* Hübner, 1793: 12. TL: Europe. Syntype(s) (unknown sex): Unknown.

*Epiblema foenella* form *trapezoidalis* Popescu-Gorj, 1965: 166. TL: Romania. Holotype (male): MGAB.

*Epiblema foenella* form *unicolor* Uffeln, 1912: 136. TL: Europe. Holotype (unknown sex): Unknown.

*Grapholitha foenella* ab. *unicolorana* Klemensiewicz, 1900: 187. TL: Europe. Syntype(s) (unknown sex): ISEZ.

**Material examined.** 1 ♂, Hualien Co., Kuyuan, Taroko Gorge, 750 m, 6-7-VII-2005, J. Heppner. [MGCL].

**Host plants.** Asteraceae: *Artemisia argyi* Lévl. *et* Vant., *A. vulgaris* L., *Centaurea* sp., *Cirsium arvense* (L.) Scop.; Poaceae: *Phragmites communis* Trin. The last seems somewhat unlikely.

**Distribution.** China (Anhui, Fujian, Gansu, Guangxi, Guizhou, Hebei, Heilongjiang, Henan, Hubei, Hunan, Jiangsu, Jiangxi, Jilin, Inner Mongolia, Qinghai, Shandong, Shaanxi, Sichuan, Taiwan, Tianjin, Xinjiang, Yunnan, Zhejiang), Central Asia, India, Japan, Kazakhstan, Mongolia, Russia (Far East), South Korea and Thailand.

### 3.6. Epinotia *Hübner, [1825]1816*

*Acalla* Hübner, [1825]1816: 383. Type species: *Tortrix ophthalmicana* Hübner, [1796,1797,1798,1799].

*Astatia* Hübner, [1825]1816: 377. Type species: *Tortrix parmatana* Hübner, [1814,1815,1816,1817].

*Asthenia* Hübner, [1825]1816: 381. Type species: *Tortrix pygmaeana* Hübner, [1796,1797,1798,1799].

*Cartella* Guenée, 1845: 174. Type species: *Tortrix cretaceana* Frölich, 1828.

*Ccalla* Caradja, 1916: 86. [misspelling of *Acalla*].

*Curtella* Stainton, 1859: 201. [misspelling of *Cartella*].

*Epinotia* Hübner, [1825]1816: 377.

*Epinotis* Kearfott, 1907: 51. [misspelling of *Epinotia*].

*Evertia* Matsumura, 1931: 1070. [misspelling of *Evetria*].

*Evetria* Hübner, [1825]1816: 378. Type species: *Tortrix piceana* Hübner, [1796,1797,1798,1799].

*Griselda* Heinrich, 1923: 186. Type species: *Griselda radicana* Heinrich, 1923.

*Hamuligera* Obraztsov, 1946: 31. Type species: *Phalaena trimaculana* Donovan, [1806].

*Hikagehamakia* Oku, 1974: 115. Type species: *Hikagehamakia albiguttata* Oku, 1974.

*Hypermecia* Guenée, 1845: 173. Type species: *Tortrix augustana* Hübner, [1811,1812,1813].

*Lithographia* Stephens, 1852: 32. Type species: *Phalaena nisella* Clerck, 1759.

*Neurasthenia* Pierce et Metcalfe, 1922: 65. Type species: *Tortrix pygmaeana* Hübner, [1796,1797,1798,1799].

*Paedisca* Treitschke, 1830: 188. Type species: *Tortrix parmatana* Hübner, [1814,1815,1816,1817].

*Pamplasia* Desmarest, 1857: 224. [misspelling of *Pamplusia*].

*Pamplusia* Guenée, 1845: 180. Type species: *Coccyx monticolana* Duponchel, 1842.

*Panoplia* Hübner, [1825]1816: 293. Type species: *Tortrix augustana* Hübner, [1811,1812,1813].

*Paragrapha* Sodoffsky, 1837: 92. [unnecessary replacement name for *Paedisca*].

*Phlaeodes* Guenée, 1845: 172. Type species: *Tortrix frutetana* Hübner, [1823,1824].

*Podisca* Guenée, 1845: 174. [misspelling of *Paedisca*].

*Poecilochroma* Stephens, 1829: 47. Type species: *Phalaena (Tortrix) solandriana* Linnaeus, 1758.

*Proteopteryx* Walsingham, 1879: 68. Type species: *Proteopteryx emarginana* Walsingham, 1879.

*Steganoptera* Herrich-Schäffer, 1851: 133. [misspelling of *Steganoptycha*].

*Steganoptycha* Stephens, 1829: 47. Type species: *Pyralis boeberana* Fabricius, 1787.

Type species: *Phalaena (Tortrix) similana* Hübner, 1793.

#### 3.6.1. *Epinotia bicolor* (Walsingham, 1900)

*Pelatea bicolor* Walsingham, 1900: 335 TL: Japan/India, Assam, Naga Hills. Syntypes (male, female): NHMUK.

*Epinotia bicolor* (Walsingham): Kawabe, 1982: 123.

**Material examined.** 1 ♂, 1 ♀, Nantou Hsien, Nanshanshi, 25-VII-1983, A. Kawabe. [USNM].

1 ♀, Nantou Co., 15 km E of Puli, 700 m, 6-V-1989, J. Heppner & H. Wang; 1 ♀, Ilan Co., 5 km N. Nanao, 100 m, 11-13-X-1984, J. Heppner & H. Wang; 1 ♂, Taitung Co., Bishan Hot Spgs., 720 m, 26-29-X-1984, J. Heppner & H. Wang. [MGCL]

**Host plants.** Fagaceae: *Quercus serrata* Thunb., *Q. acutissima* Carruth., *Q. phillyreoides* A. Gray, *Q. glauca* Thunb.; Pinaceae: *Pinus pumila* (Pall.) Regel. The last, reported by Suzuki & Komai (1984), is unusual.

**Distribution.** China (Fujian, Gansu, Gui-zhou, Hebei, Henan, Hubei, Hunan, Shaanxi, Sichuan, Taiwan, Tianjin), Korea Japan, India and Vietnam.

#### 3.6.2. *Epinotia fujisawai* Kawabe, 1993

*Epinotia fujisawai* Kawabe, 1993: 237. TL: Taiwan, Nantou Hsien, Lushan Spa. Holotype (male): USNM.

**Material examined.** Holotype: ♂, Nantou Hsien, Lushan spa, 1200 m, 7-8-VI-1988, K. Fujisawa. Paratypes: 2 ♀♀, Hualien Hsien, Tayuling, 2560 m, 12-VI-1988, K. Fujisawa. [USNM].

1 ♂, Hualien Hsien, Tayuling, 2560 m, 12-VI-1988, K. Fujisawa; 1 ♀, Nantou Co., Tayuling, 2500 m, 9-18-VI-1980, D. R. Davis. [USNM].

**Distribution.** China (Taiwan).

#### 3.6.3. *Epinotia majorana* (Caradja, 1916)

*Gypsonoma majorana* Caradja, 1916: 61. TL: Russia, Far East, Khabarovsky Krai, Radde. Lectotype: MGAB.

*Eucosma leucantha* Meyrick, 1931: 145. TL: Japan, Honshu, Tokyo Prefecture. Holotype (female): NHMUK.

*Epinotia leucantha* Issiki, 1957: 59.

*Epinotia majorana* (Caradja), Razowski, 1971: 521.

**Material examined.** 1 ♀, Taihoku, E-III-1927, S. Issiki; 1 ♂, Taihoku, VI-1921, S. Issiki; 1 ♀, Taihoku, 6-IV-1946, S. Issiki; 1 ♀, Kao Hsiung Co., 1–2 km W Meishan, 870 m, 29-VI-2-VII-1980, D. R. Davis. [USNM]. (Adult Number: Sun-153 ♂, Genitalia Slide Numbers: USNM144429 ♂, USNM144430 ♀)

**Host plants.** Apiaceae: *Daucus carota* L., *Heracleum moellendorfii* Hance; Betulaceae: *Betula dahurica* Pall.

**Distribution.** China (Taiwan), Japan, Korea and Russia.

**Remarks.** This species is recorded for the first time in Taiwan.

#### 3.6.4. *Epinotia melanosticta* (Wileman *et* Stringer, 1929)

*Eucosma melanosticta* Wileman *et* Stringer, 1929: 67. TL: Formosa [Taiwan], Arizan. Holotype (male): NHMUK.

*Epinotia melanosticta* (Wileman *et* Stringer): Kawabe, 1992: 107.

**Material examined.** 4 ♂♂, Nantou Co., Tayuling, 2500 m, 9-18-VI-1980, D. R. Davis; 4 ♂♂, Nantou Co., Mei-feng, 30 km S Tayuling, 2200 m, 1-8-VI-1980, D. R. Davis; 1 ♀, Hassenzan, 6-VI-1942, A. Mutuura. [USNM].

1 ♂, Taiwan: Hassenzan, 6-VI-1942, A. Mutuura; 2 ♂♂, Nantou Co., Tayuling, 2570 m, 15-18-VI-1982, J. Heppner; 1 ♂, Hualien Co., Kuyuan, Taroko Gorge, 750 m, 6-7-VII-2005, J. Heppner. [MGCL].

**Distribution.** China (Taiwan).

#### 3.6.5. *Epinotia monticola* Kawabe, 1993

*Epinotia monticola* Kawabe, 1993: 238. TL: Taiwan, Hualien Hsien, Hohuanshan. Holotype (male): USNM.

**Material examined.** Holotype: ♂, Hualien Hsien, Hohuanshan, 3100 m, 10-11-VI-1988, K. Fujisawa. [USNM].

**Distribution.** China (Taiwan).

#### 3.6.6. *Epinotia piceicola* Kuznetzov, 1970

*Epinotia piceicola* Kuznetzov, 1970: 437. [replacement name for *piceae*]

*Epinotia (Steganoptycha) piceae* Kuznetzov, 1968: 569. TL: Russia, Kuril Islands, Kunashir Island, near Sernovodsk. Holotype (male): ZISP. [preoccupied].

**Host plant.** Pinaceae: *Abies mariesii* Mast., *Abies sachalinensis* (F. Schmidt) Mast., *Picea glehnii* (F. Schmidt) Mast., *Picea jezoensis* (Siebold *et* Zucc.) Carriere.

**Distribution.** China (Taiwan), Japan and Russia.

#### 3.6.7. *Epinotia rasdolnyana* (Christoph, 1881)

*Steganoptycha rasdolnyana* Christoph, 1881: 427. TL: Russia, Primorsky Krai, Vladi-vostok. Syntype(s) (unknown sex): Unknown.

*Epinotia rasdolniana* (Christoph): Kennel, 1916: 498. [misspelling of *rasdolnyana*].

*Epinotia rasdolnyana* (Christoph): Issiki, 1957: 59.

**Material examined.** 1 ♂, 8 ♀♀, Nantou Hsien, Tsuifeng, 2400 m, 29-XII-1989, A. Kawabe; 1 ♂, Chiai Hsien, Alishan, 2270 m, 3-I-1986, H. Nakajima. [USNM].

**Host plants.** Aceraceae: *Acer pictum* Thunb. *et* Murray, *A. ukurunduense* Trautv. *et* Mey.

**Distribution.** China (Northeast Part, Taiwan), Japan, Korea and Russia.

#### 3.6.8. *Epinotia rubricana* (Kuznetzov, 1968)

*Epinotia (Steganoptycha) rubricana* Kuznetzov, 1968: 575. TL: Russia, Primorsky Krai, near Vladivostok. Holotype (female): ZISP.

*Epinotia rubricana* (Kuznetzov): Kawabe, 1970: 204.

**Material examined.** 3 ♂♂, 2 ♀♀, Nantou Hsien, Lushan spa, 1200 m, 27-29-VII-1983, A. Kawabe; 4 ♂♂, 2 ♀♀, Nantou Co., Tayuling, 2500 m, 9-18-VI-1980, D. R. Davis; 2 ♂♂, Nantou Hsien, Tsingjing Farm, 1900 m, 9-VI-1988, K. Fujisawa; 2 ♂♂, Nantou Hsien, Lushan spa, 1200 m, 3-5-VIII-1985, A. Kawabe; 1 ♂, Nantou Hsien, Lushan spa, 1200 m, 7-8-VI-1988, K. Fujisawa; 1 ♀, Hualien Hsien, Wenshan spa, 580 m, 4-5-VIII-1983, A. Kawabe; 1 ♀, Funki-ko, 26-VIII-1968, M. Saikawa; 1 ♀, Rozan spa, Formosa, 27-VIII-1969, H. Nakajima; 1 ♀, Hualien Hsien, Kuanyuan, 2400 m, 7-8-VIII-1987, A. Kawabe; 1 ♂, (Lishan) Taichung Hsien, Formosa, 9-VIII-1974, Y. Kishida; 1 ♂, Syorei, 12-VIII-1948, A. Mutuura; 1 ♂, Tattaka, 19-VII-1925, S. Issiki; 1 ♂, S. Issiki; 1 ♂, Taihoku, 1-VI-1921, S. Issiki. [USNM].

1 ♂, 4 ♀♀, Hsinchu Co., Kuangwu For. Sta., 2000 m, 18-25-VIII-1988, J. Heppner & H. Wang; 8 ♂♂, Hsinchu/Miaoli Kuangwu, 2000 m, 24-25-VI-1985, J. Heppner & H. Wang; 1 ♂, Nantou Co., Tayuling, 2570 m, 10-14-VI-1982, J. Heppner; 2 ♂♂, Hualien Co., Kuyuan, Taroko Gorge, 750 m, 6-7-VII-2005, J. Heppner; 1 ♀, Taoyuan Co., Upper Palin, 1350 m, 4-5-VI-2005, J. Heppner & H. Wang; 46 ♂♂, 26 ♀♀, Kaohsiung Co., Tienchih, 2210 m, 24-X-1984, J. Heppner & H. Wang; 28 ♂♂, 15 ♀♀, Taichung Co., Chingshan, 2750 m, 25-X-1984, J. Heppner & H. Wang; 1 ♂, Kaohsiung Co., Tienchih, Yushan N.P., 2280 m, 7-VII-1996, J. Heppner & H. Wang. [MGCL].

**Distribution.** China (Taiwan), Japan, Korea and Russia.

#### 3.6.9. *Epinotia salicicolana* Kuznetzov, 1968

*Epinotia salicicolana* Kuznetzov, 1968: 577. TL: Russia, Kuril Islands, Kunashir, near Sernovodsk. Holotype (male): ZISP.

**Host plants.** Salicaceae: *Salix caprea* L., *S. sachalinensis* Fr. Schm.

**Distribution.** China (Shaanxi, Taiwan), Japan and Russia.

### 3.7. Eucosma *Hübner, 1823*

*Eucosma* Hübner, 1823: 28. Type species: *Tortrix circulana* Hübner, 1823.

*Catoptria* Guenée, 1845: 187. Type species: *Phalaena pupillana* Clerck, 1759. [preoccupied].

*Calosetia* Stainton, 1859: 271. Type species: *Tortrix nigromaculana* Haworth, 1811[1812].

*Pygolopha* Lederer, 1859: 123. Type species: *Pygolopha trinacriana* Lederer, 1859.

*Affa* Walker, 1863: 202. Type species: *Affa bipunctella* Walker, 1863.

*Exentera* Grote, 1877: 227. Type species: *Exentera aprilana* Grote, 1877.

*Exenterella* Grote, 1883: 23. [unnecessary replacement name for *Exentera*].

*Palpocrinia* Kennel, 1919: 66. Type species: *Palpocrinia ottoniana* Kennel, 1919.

*Ascelodes* Fletcher, 1929: 25. [nomen nudum].

#### 3.7.1. *Eucosma notanthes* Meyrick, 1936

*Eucosma notanthes* Meyrick, 1936: 610. TL: Taiwan, Kagi. Holotype (female): NHMUK.

*Cydia notanthes* (Meyrick): Kawabe, 1992: 108.

**Material examined.** 1 ♂, Kagi, 20-IV-1931, T. Shiraki. [USNM].

1 ♂, Kaohsiung Co., Liukuei, Shanpin For., 750 m, 29-IV/3-V-1989, J. Heppner & H. Wang; 1 ♀, Pingtung Co., Kenting Park, 255 m, 23-28-IV-1989, J. Heppner & H. Wang; 1 ♀, Ilan Co., Chilan Nsy., Tuchan, 480 m, 15-17-X-1984, J. Heppner & H. Wang; 1 ♀, Pingtung Co., Kenting Park, 50 m, 1-5-IX-1983, J. Heppner; 2 ♂♂, 2 ♀♀, Hualien Co., Tien Hsiang, 435 m, 25-VIII-1983, J. Heppner. [MGCL].

**Host plants.** Elaeocarpaceae: *Elaeocarpus serratus* L.; Oxalidaceae: *Averrhoa carambola*. This species, known as the carambola or starfruit borer, is of quarantine significance.

**Distribution.** China (Taiwan) and Japan.

#### 3.7.2. *Eucosma pentagonaspis* Meyrick, 1931

*Eucosma pentagonaspis* Meyrick, 1931: 144. TL: China, Taiwan. Holotype (male): NHMUK.

**Distribution.** China (Taiwan).

### 3.8. Fibuloides *Kuznetzov, 1997*

*Fibuloides* Kuznetzov, 1997. Type species: *Fibuloides modificana* Kuznetzov, 1997.

#### 3.8.1. *Fibuloides corinthia* (Meyrick, 1912)

*Acroclita corinthia* Meyrick, 1912: 858. TL: Sri Lanka, Maskeliya. Lectotype (male): NHMUK.

*Fibuloides corinthia* (Meyrick): Horak, 2006: 330.

*Fibuloides nigrovenana* (Kuznetzov): Horak, 2006: 330.

**Material examined.** 8 ♂♂, 12 ♀♀, Chiai Hsien, Fenchihu, 1400 m, 11-12-VIII-1985, A. Kawabe; 2 ♂♂, 9 ♀♀, Nantou Hsien, Lushan spa, 1200 m, 3-5-VIII-1985, A. Kawabe; 2 ♂♂, Nantou Hsien, Lushan spa, 1200 m, 9-10-VIII-1987, A. Kawabe; 1 ♀, Chiai Hsien, Alishan, 2270 m, 8-10-VIII-1985, A. Kawabe; 1 ♂, Hualien Hsien, Hungyeh spa, 200 m, 29-30-III-1984, A. Kawabe; 1 ♂, Miaoli Hsien, Hernglong Lodge, 550 m, nr Taian spa, 550 m, 8-VIII-1983, A. Kawabe; 1 ♀, Hualien Hsien, Wenshan spa, 580 m, 4-5-VIII-1983, A. Kawabe; 1 ♂, Kagi, 11-IV-1935, S. Issiki; 1 ♀, Kagi, 24-X-1934, S. Issiki; 1 ♂, Nantou Hsien, Lushan spa, 1200 m, 7-8-VI-1988, K. Fujisawa; 1 ♂, Nantou Hsien, Lushan spa, 1200 m, 3-5-VIII-1985, A. Kawabe; 1 ♂, Chiai Hsien, Fenchihu, 1400 m, 11-12-VIII-1985, A. Kawabe; 1 ♂, Nantou Hsien, Wushe, 1150 m, 1-V-1990, A. Kawabe; 1 ♂, Nantou Hisen, Lushan Spa, 1250 m, 30-IV-1990, A. Kawabe. [USNM].

1 ♂, Taitung Co., Yakou, 2750 m, 25-X-1984, J. Heppner & H. Wang; 2 ♂♂, 13 ♀♀, Taichung Co., Chingshan, 1100 m, 31-VIII/4-IX-1988, J. Heppner & H. Wang; 1 ♀, Hualien County, Tien Shien, 220 m, 5-7-VIII-1992, J. Heppner; 1 ♀, Hsinchu/Miaoli Kuangwu, 2000 m, 24-25-VI-1985, J. Heppner & H. Wang; 1 ♂, 1 ♀, Taichung Co., Chingshan, 1100 m, 8-11-V-1989, J. Heppner & H. Wang; 1 ♂, Taitung Co., Bishan Hot Spgs., 720 m, 26-29-X-1984, J. Heppner & H. Wang; 1 ♂, Nantou Co., 5 km N. Chitou, 500 m, 29-VI-1985, J. Heppner & H. Wang; 1 ♂, Kaohsiung Co., Lukuei For. Sta., 750 m, 29-IV/3-V-1989, J. Heppner & H. Wang; 1 ♀, Ilan Co., Tuchan, 480 m, 1-2-VII-1982, J. Heppner; 3 ♀♀, Taitung Co., Bishan Hot Spgs., 720 m, 26-29-X-1984, J. Heppner & H. Wang; 2 ♀♀, Ilan Co., Chilan Nsy., Tuchan, 480 m, 15-17-X-1984, J. Heppner & H. Wang; 1 ♀, Nantou Co., Meifeng, 2100 m, 6-9-IX-1988, J. Heppner; 1 ♀, Ilan Co., Taipingshan, 1900 m, 13-V-1989, J. Heppner & H. Wang. [MGCL].

**Host plant.** Sapindaceae: *Litchi chinensis* Sonn.

**Distribution.** China (Taiwan), Japan, Sri Lanka and Vietnam.

#### 3.8.2. *Fibuloides japonica* (Kawabe, 1978)

*Eucoenogenes japonica* Kawabe, 1978: 185. TL: Japan, Honshu, Gunma Prefecture, Mt. Mikaboyama. Holotype (male): USNM.

*Fibuloides japonica* (Kawabe): Horak 2006: 330.

**Material examined.** 1 ♂, (Suchungchi) Pingtung-Hsien, 10-VIII-1971, Y. Shibata; 1 ♂, Nantou Hsien, Nanshanchi, 25-26-VII-1983, A. Kawabe. [USNM].

2 ♂♂, Nantou Co., Tungpu, 1150 m, 1-VII-1985, J. Heppner & H. Wang. [MGCL].

**Distribution.** China (Anhui, Fujian, Guizhou, Henan, Hubei, Shaanxi, Sichuan, Taiwan, Zhejiang), Japan and Korea.

### 3.9. Gibberifera *Obraztsov, 1946*

*Gibberifera* Obraztsov, 1946: 35. Type species: *Penthina simplana* Fischer von Roslerstamm, 1836.

*Gibberelifera* Byun *et* Park, 1994: 163. [misspelling of *Gibberifera*].

#### 3.9.1. *Gibberifera glaciata* (Meyrick, 1907)

*Cydia glaciata* Meyrick, 1907: 143. TL: India, Assam, Khasi Hills. Lectotype (male): NHMUK.

*Eucosma glaciata* (Meyrick): Clarke, 1958: 364.

Gibberifera simplana glaciata: Diakonoff, 1964: 24.

*Gibberifera glaciata* (Meyrick): Kawabe *et* Nasu, 1994: 85.

**Material examined.** 3 ♀♀, Tayuling, Hualien Hsien, 28-VII-1985, Y. Kishida; 1 ♀, Hualien Hsien, Kuanyuan, 2400 m, 7-8-VIII-1987, A. Kawabe; 1 ♀, Hualien Hsien, Wenshan spa, 580 m, 4-5-VIII-1983, A. Kawabe; 1 ♂, Nantou Co., Tayuling, 2500 m, 9-18-VI-1980, D. R. Davis. [USNM].

1 ♂, Nantou Co., Tayuling, 2570 m, 10-14-VI-1982, J. Heppner; 1 ♂, Nantou Co., Meifeng, 2100 m, 6-9-IX-1988, J. Heppner; 1 ♂, Nantou Co., Meifeng, 2100 m, 6-9-IX-1988, J. Heppner. [MGCL].

**Host plants.** Salicaceae: *Populus ciliata* (Wall.) *et* Royle, *Salix* sp.

**Distribution.** China (Guizhou, Henan, Hunan, Sichuan, Taiwan, Xizang, Yunnan), India, Nepal, Pakistan and Thailand.

#### 3.9.2. *Gibberifera simplana* (Fischer Von Röslerstamm, 1835)

*Penthina simplana* Fischer von Röslerstamm, 1836: 38. TL: Czech Republic, near Reichstadt. Holotype (unknown sex): Unknown.

*Gibberifera simplana* (Fischer von Röslerstamm): Obraztsov, 1946: 26.

**Material examined.** 1 ♀, Taiwan: Pianan-anbu, 10-VIII-1943, A. Mutuura. [USNM].

1 ♀, Nantou Co., Meifeng, 2100 m, 6-9-IX-1988, J. Heppner. [MGCL].

**Host plants.** Salicaceae: *Populus tremula* L., *Salix* sp.

**Distribution.** China (Gansu, Hebei, Henan, Hubei, Hunan, Jilin, Shaanxi, Taiwan), Europe, Japan, Korea and Russia.

### 3.10. Gypsonoma *Meyrick, 1895*

*Gypsonoma* Meyrick, 1895: 481. Type species: *Tortrix dealbana* Frölich, 1828.

#### *Gypsonoma attrita* Falkovitsh, 1965

*Gypsonoma attrita* Falkovitsh, 1965: 431. TL: Russia, Primorsky Krai, Ussuriysk. Holotype (male): ZISP.

**Material examined.** 1 ♂, [Mount] Arisan, X-1925, S. Issiki; 1 ♂, Musha, 18-V-15-VI-1919, T. Okuni, J. Sonan; 1 ♀, Tattaka, 8-VII-1943, S. Issiki; 7 ♂♂, 6 ♀♀, Nantou Co., Tayuling, 2500 m, 9-18-VI-1980, D. R. Davis; 1 ♂, Chiai Hsien, Alishan, 2270 m, 8-10-VIII-1985, A. Kawabe; 1 ♀, Hualien Hsien, Hohuanshan, 3100 m, 30-VII-1-VIII-1983, A. Kawabe. [USNM]. 3 ♂♂, 1 ♀, Nantou Co., Tayuling, 2570 m, 5-9-VI-1982, J. Heppner; 4 ♂♂, Nantou Co., Tayuling, 2570 m, 10-14-VI-1982, J. Heppner. [MGCL].

**Distribution.** China (Taiwan), Japan and Russia.

### 3.11. Heleanna *Clarke, 1976*

*Heleanna* Clarke, 1976: 11. Type species: *Rhopobota physalodes* Meyrick, 1910.

#### *Heleanna melanomochla* (Meyrick, 1936)

*Acroclita melanomochla* Meyrick, 1936: 24. TL: Formosa [Taiwan], Heito. Lectotype (male): NHMUK.

*Heleanna melanomochla* (Meyrick): Brown, 2005: 377.

**Material examined.** 1 ♂, Suiryuto, 12-X-1935, S. Issiki; 1 ♂, Taihoku, 18-V-1935, S. Issiki; 1 ♂, Heito, 11-IV-1935, S. Issiki; 1 ♂, Heito, 14-IV-1935, S. Issiki; 1 ♂, Taihoku, 20-X-1935, S. Issiki. [USNM].

3 ♂♂, 3 ♀♀, Taipei Co., Wulai, 200 m, 17-19-VI-1985, J. Heppner & H. Wang; 1 ♂, Ilan Co., Mingchyr Rec. Area, 1250 m, 9-10-VII-1996, J. Heppner & H. Wang. [MGCL].

**Host plant.** Anacardiaceae: *Mangifera indica* L.

**Distribution.** China (Taiwan).

### 3.12. Hendecaneura *Walsingham, 1900*

*Hendecaneura* Walsingham, 1900: 401. Type species: *Hendecaneura impar* Walsingham, 1900.

*Eucosmodes* Kuznetzov, 1973: 689. Type species: *Eucosma axiotima* Meyrick, 1937.

#### *Hendecaneura axiotima* (Meyrick, 1937)

*Eucosma axiotima* Meyrick, 1937: 178. TL: China, Yunnan Province, Likiang. Lectotype (male): NHMUK.

*Hendecaneura axiotima* (Meyrick): Razowski, 1989: 289.

**Material examined.** 1 ♂, Tayuling, Hualien Hsien, A-X-1926, Y. Kishida; 1 ♂, Nantou Co., Tayuling, 2500 m, 9-18-VI-1980, D. R. Davis; 1 ♀, Mt. Foufuanshan, 3100 m, 8-VIII-1974, Y. Kishida. [USNM].

2 ♀♀, Taitung Co., Yakou, 2750 m, 25-X-1984, J. Heppner & H. Wang. [MGCL].

**Distribution.** China (Sichuan, Taiwan, Yunnan, Xizang), India and Nepal.

### 3.13. Hermenias *Meyrick, 1911*

*Hermenias* Meyrick, 1911: 225. Type species: *Hermenias epidola* Meyrick, 1911.

*Herninias* Lower, 1896. [misspelling of *Hermenias*].

#### 3.13.1. *Hermenias pilushina* Razowski, 2000

*Hermenias pilushina* Razowski, 2000: 326. TL: China, Taiwan. Holotype (male): CMNH.

**Distribution.** China (Taiwan).

**Remarks.** This species was described by Razowski (2000) [7] based on a single male deposited in the CMNH.

#### 3.13.2. *Hermenias semicurva* (Meyrick, 1912)

*Eucosma semicurva* Meyrick, 1912: 864. TL: India, Assam, Khasi Hills. Lectotype (female): NHMUK.

**Material examined.** 1 ♂, Tayuling, Hualien Hsien, 28-VII-85, Y. Kishida. [USNM].

1 ♂, Hsinchu Co., Kuangwu For. Sta., 2000 m, 18-25-VIII-1988, J. Heppner & H. Wang. [MGCL]. (Adult Number: TORT12084 ♂, Genitalia Slide Number: YH-Sun FL188 ♂)

**Distribution.** China (Taiwan) and India.

**Remarks.** This species is recorded for the first time in China.

### 3.14. Kennelia *Rebel, 1901*

*Kennelia* Rebel, 1901. Type species: *Anomalopteryx xylinana* Kennel, 1901.

*Anomalopteryx* Kennel, 1900. Preoccupied.

#### *Kennelia albifacies* (Walsingham, 1900)

*Lipsotelus albifacies* Walsingham, 1900: 570. TL: India. Holotype (unknown sex): OUM.

*Argyroploce corthyntis* Meyrick, 1909: 596.

*Kennelia albifacies* (Walsingham): Diakonoff, 1975: 312.

**Distribution.** China (Taiwan), India, and Sri Lanka.

### 3.15. Lepteucosma *Diakonoff, 1971*

*Lepteucosma* Diakonoff, 1971: 179. Type species: *Lepteucosma oxychrysa* Diakonoff, 1971.

*Ceriodes* Diakonoff, 1984: 374. Type species: *Eucosma ceriodes* Meyrick, 1909. [subgenus of *Epinotia*].

#### 3.15.1. *Lepteucosma blandoides* Lu *et* Hsu, 2024

*Lepteucosma blandoides* Lu *et* Hsu, 2024: 2. TL: Taiwan, Nantou. Holotype (male): NMNS.

**Distribution.** China (Taiwan).

#### 3.15.2. *Lepteucosma ceriodes* Meyrick, 1909

Eucosma ceriodes Meyrick, 1909: 607. TL: India, Assam, Khasi Hills. Lectotype (male): NHMUK.

Lepteucosma ceriodes (Meyrick): Diakonoff, 1971: 180.

*Epinotia ceriodes*: Heppner, 2012: 19.

**Host plant.** Rosaceae: *Rubus* sp.

**Distribution.** China (Sichuan, Taiwan, Zhejiang), India and Vietnam.

#### 3.15.3. *Lepteucosma dasyueshanensis* Lu *et* Hsu 2024

*Lepteucosma dasyueshanensis* Lu *et* Hsu, 2024: 6. TL: Taiwan, Miaoli. Holotype (male): NMNS.

**Distribution.** China (Taiwan).

#### 3.15.4. *Lepteucosma shikokuensis* (Kawabe, 1984)

*Epinotia shikokuensis* Kawabe, 1984: 187. TL: Japan, Honshu, Shikoku, Kagawa Prefecture, Oku-shio-iri. Holotype (male): USNM.

*Lepteucosma shikokuensis* (Kawabe): Kuznetzov, 1988: 84.

**Material examined.** Paratypes: 1 ♂, Hitotsu-uchi, Shionoe, Kagawa, 22-IX-1973, H. Toshima; 1 ♀, Oku-shio-iri, Chunan, Kagawa, 2-IX-1972, H. Toshima. [USNM].

13 ♂♂, 2 ♀♀, Nantou Hsien, Lushan spa, 1200 m, 27-29-VII-1983, A. Kawabe; 8 ♂♂, Nantou Hsien, Nanshanchi, 25-26-VII-1983, A. Kawabe; 2 ♂♂, Miaoli Hsien, Hernglong Lodge, 550 m, nr. Taian spa, 8-VIII-1983, A. Kawabe; 1 ♂, Hualien Hsien, Wenshan spa, 580 m, 4-5-VIII-1983, A. Kawabe; 2 ♂♂, Nantou Hsien, Lushan spa, 1200 m, 3-5-VIII-1985, A. Kawabe; 2 ♂♂, Taoyuan Hsien, Palin, 1000 m, 31-VII-2-VIII-1985, A. Kawabe; 1 ♂, Nantou Hsien, Lushan spa, 1200 m, 9-10-VIII-1987, A. Kawabe; 1 ♀, Nantou Hsien, Yushih, 1700 m, 3-4-VIII-1987, A. Kawabe; 1 ♂, Nantou Hsien, Wushe, 1150 m, 1-V-1990, A. Kawabe; 1 ♀, Marreba, 13-VIII-1943, A. Mutuura. [USNM].

4 ♂♂, 2 ♀♀, Taoyuan Co., Upper Palin, 1350 m, 4-5-VI-2005, J. Heppner; 2 ♂♂, Taichung Co., Chingshan, 1100 m, 8-11-V-1989, J. Heppner & H. Wang; 1 ♂, Taitung Co., Bishan Hot Spgs., 720 m, 26-29-X-1984, J. Heppner & H. Wang; 3 ♂♂, Taichung Co., Chingshan, 1100 m, 31-VIII/4-IX-1988, J. Heppner & H. Wang; 2 ♂♂, 1 ♀, Taoyuan Co., Upper Palin, 1500 m, 7-9-VII-1985, J. Heppner & H. Wang; 1 ♂, Taichung Co., Chinn, 1100 m, 27-III/1-IV-1990, J. Heppner & H. Wang; 1 ♂, Taoyuan Co., Upper Palin, 1350 m, 4-5-VII-2005, J. Heppner. [MGCL].

**Distribution.** China (Fujian, Guizhou, Taiwan) and Japan.

### 3.16. Melanodaedala *Horak, 2006*

*Melanodaedala* Horak, 2006. Type species: *Bathrotoma scopulosana* Meyrick, 1881.

#### *Melanodaedala melanoneura* (Meyrick, 1912)

*Eucosma melanoneura* Meyrick, 1912: 886. TL: India, Assam, Khasi Hills. Lectotype (male): NHMUK.

*Megaherpystis melanoneura* (Meyrick): Kuznetzov, 1988: 81.

*Melanodaedala melanoneura* (Meyrick): Horak, 2006: 320.

**Material examined.** 1 ♀, Nantou Hsien, Lushan spa, 1200 m, 3-5-VIII-1985, A. Kawabe; 1 ♀, Hualien Hsien, Wenshan spa, 580 m, 4-5-VIII-1983, A. Kawabe; 1 ♂, Miaoli Hsien, Hernglong Lodge, 550 m. nr. Taian spa, 550 m, 8-VIII-1983, A. Kawabe; 1 ♂, Oomura, Chichi-jima, 24-IV-1973, Y. Kusui; 1 ♀, Taihoku, 18-X-1923, S. Issiki; 1 ♂, Kagi, 15-IV-1935, S. Issiki; 1 ♂, Raisya, 24-XI-1934, S. Issiki; 2 ♂♂, Kaohsiung Co., 10-11 km NE Chiahsien, ca. 300 m, 3-8-VII-1980, D. R. Davis. [USNM].

1 ♀, Taipei Co., Yangmingshan, 450 m, 20-21-VII-2005, J. Heppner; 1 ♀, Taipei Co., Wulai, 200 m, 17-19-VI-1985, J. Heppner & H. Wang; 1 ♂, Taoyuan Co., Upper Palin, 1350 m, 4-5-VII-2005, J. Heppner; 1 ♂, Taichung Co., Chingshan, 1100 m, 27-III/1-IV-1990, J. Heppner & H. Wang. [MGCL].

**Host plants.** Anacardiaceae: *Mangifera indica* L., *Rhus chinensis* Mill.

**Distribution.** China (Anhui, Fujian, Guangdong, Guangxi, Guizhou, Henan, Hubei, Hunan, Shaanxi, Shandong, Sichuan, Taiwan), India, Japan, Korea and Vietnam.

### 3.17. Notocelia *Hübner, [1825]1816*

Notocelia Hübner, [1825]1816: 379. Type species: Phalaena (Tortrix) uddmanniana Linnaeus, 1758.

*Aspis* Treitschke, 1829: 231. Type species: *Phalaena (Tortrix) solandriana* Linnaeus, 1758 *sensu* Treitschke, 1829 [=*Phalaena (Tortrix) uddmanniana* Linnaeus, 1758]. [preoccupied].

*Aspidia* Duponchel, 1835: 176. [replacement name for *Aspis*].

*Pardia* Guenée, 1845: 155. Type species: *Tortrix tripunctana* Denis et Schiffermüller, 1775 [=*Phalaena (Tinea) cynosbatella* Linnaeus, 1758].

*Bardia* Obraztsov, 1965: 371. [misspelling of *Pardia*].

#### 3.17.1. *Notocelia donaldana* Kawabe, 1993

*Notocelia donaldana* Kawabe, 1993: 238. TL: Taiwan, Nantou Hsien, Mei-feng, 30 km S Tayuling. Holotype (male): USNM.

**Material examined.** Holotype: ♂, Nantou Co., Mei-feng, 30 km S Tayuling, 2200 m, 1-8-VI-1980, D. R. Davis. [USNM].

2 ♂♂, Nantou Co., Mei-feng, 30 km S Tayuling, 2200 m, 1-8-VI-1980, D. R. Davis; 1 ♂, Hassenzan, 6-VI-1942, S. Issiki, Issiki Collection, 1972; 1 ♂, Tattaka, 19-VII-1925, S. Issiki, Issiki Collection, 1972. [USNM].

**Distribution.** China (Taiwan).

#### 3.17.2. *Notocelia kurosawai* Kawabe, 1986

*Notocelia kurosawai* Kawabe, 1986: 80. TL: Taiwan, Hualien Hsien, Hohuanshan. Holotype (male): USNM.

**Material examined.** Holotype: ♂, Hualien Hsien, Hohuanshan, 3100 m, 30-VII-1-VIII-1983, A. Kawabe. Paratypes: 5 ♂♂, 1 ♀, Hualien Hsien, Hohuanshan, 3100 m, 30-VII-1-VIII-1983, A. Kawabe. [USNM].

11 ♂♂, 2 ♀♀, Nantou Co., Tayuling, 2500 m, 9-18-VI-1980, D. R. Davis; 1 ♂, Nantou Co., Mei-feng, 30 km S Tayuling, 2200 m, 1-8-VI-1980, D. R. Davis; 1 ♂, Hualien Hsien, Hohuanshan, 3100 m, 5-6-VIII-1987, A. Kawabe; 1 ♂, Higasinoko, 3-VI-1943, S. Issiki. [USNM].

6 ♂♂, 5 ♀♀, Nantou Co., Tayuling, 2570 m, 10-14-VI-1982, J. Heppner; 1 ♂, Nantou Co., Tayuling, 2570 m, 5-9-VI-1982, J. Heppner; 1 ♂, 1 ♀, Nantou Co., Tayuling, 2570 m, 15-18-VI-1982, J. Heppner; 2 ♀♀, Hualien Co., Kuyuan, Taroko Gorge, 750 m, 6-7-VII-2005, J. Heppner. [MGCL].

**Distribution.** China (Taiwan).

### 3.18. Peridaedala *Meyrick, 1925*

*Peridaedala* Meyrick, 1925: 139.

Type species: *Peridaedala hierograpta* Meyrick, 1925.

#### 3.18.1. *Peridaedala algosa* (Meyrick, 1912)

*Spilonota algosa* Meyrick, 1912: 854. TL: India, Assam, Khasi Hills. Lectotype (male): NHMUK.

*Peridaedala algosa* (Meyrick): Brown, 2005: 435.

**Material examined.** 1 ♀, Formosa [Taiwan], III–1925, S. Issiki; 1 ♀, Nantou Hsien, Wushe, 1000 m, 6-7-VIII-1985, A. Kawabe. [USNM].

1 ♀, Nantou Co., Meifeng, 2100 m, 6-9-IX-1988, J. Heppner; 1 ♀, Taichung Co., Chingshan, 1100 m, 31-VIII/4-IX-1988, J. Heppner & H. Wang; 1 ♀, Ilan Co., Taipingshan, 1900 m, 13-V-1989; 1 ♀, Taichung Co., Chingshan, 1100 m, 27-III/1-IV-1990, J. Heppner & H. Wang. [MGCL].

**Distribution.** China (Taiwan), India and Japan.

#### 3.18.2. *Peridaedala japonica* Oku, 1979

*Peridaedala japonica* Oku, 1979: 590. TL: Japan, Honshu, Wakayama Prefecture, Mt. Iwawaki-san. Holotype (male): EIHU.

*Assulella optabilana* Kuznetzov, 1979: 80.

**Distribution.** China (Taiwan), Korea and Japan.

### 3.19. Retinia *Guenée, 1845*

*Retinia* Guenée, 1845: 180. Type species: *Phalaena (Tortrix) resinella* Linnaeus, 1758.

*Petrova* Heinrich, 1923: 21. Type species: *Retinia comstockiana* Fernald, 1879.

#### *Retinia cristata* (Walsingham, 1900)

*Enarmonia cristata* Walsingham, 1900: 439 TL: Japan. Holotype (male): NHMUK.

*Petrova insignis* Heinrich, 1928: 63. TL: Japan, Honshu, Yokohama. Holotype (male): USNM.

*Retinia cristata* (Walsingham): Issiki *et* Mutuura, 1961: 34.

*Petrova cristata* (Walsingham): Kawabe, 1982: 132.

**Material examined.** 1 ♂, Baibara, 24-III-1943, S. Issiki. [USNM].

1 ♂, Taichung Co., Chingshan, 1100 m, 31-VIII/4-IX-1988, J. Heppner & H. Wang. [MGCL].

**Host plants.** Pinaceae: *Pinus massoniana* Lamb., *P. tabulaeformis* Carr., *P. thunbergii* Parl., *P. densiflora* Sieb. *et* Zucc., *P. taiwanensis* Hayata.

**Distribution.** China (Anhui, Beijing, Guangdong, Guangxi, Hebei, Heilongjiang, Henan, Hubei, Hunan, Jiangsu, Jiangxi, Liaoning, Shaanxi, Shandong, Shanxi, Sichuan, Taiwan, Tianjin, Yunnan, Zhejiang), Japan and Korea.

### 3.20. Rhopalovalva *Kuznetzov, 1964*

*Rhopalovalva* Kuznetzov, 1964. Type species: *Eudemis lascivana* Christoph, 1882.

#### *Rhopalovalva catharotorna* (Meyrick, 1935)

*Acroclita catharotorna* Meyrick, 1935: 53. TL: China, Zhejiang, Mt. Tianmu. Lectotype (male): NHMUK.

*Rhopalovalva catharotorna* (Meyrick): Diakonoff, 1973: 692.

**Distribution.** China (Beijing, Hunan, Shanghai, Taiwan, Tianjin, Zhejiang) and Japan.

### 3.21. Rhopobota *Lederer, 1859*

*Rhopobota* Lederer, 1859: 366. Type species: *Tortrix naevana* Hübner, [1814,1815,1816,1817].

*Erinaea* Meyrick, 1907: 141. Type species: *Erinaea chlorantha* Meyrick, 1907 [=*Teras verditer* Hampson, 1891].

*Kundrya* Heinrich, 1923: 192. Type species: *Kundrya finitimana* Heinrich, 1923.

*Norma* Heinrich, 1923: 191. Type species: *Epinotia dietziana* Kearfott, 1907.

*Eumarissa* Clarke, 1976: 32. Type species: *Eumarissa leucognoma* Clarke, 1976.

*Kundyra* Razowski, 1977: 253. [misspelling of *Kundrya*].

#### 3.21.1. *Rhopobota antrifera* (Meyrick, 1935)

*Eucosma antrifera* Meyrick, 1935: 56. TL: China, Tien-Mu-Shan. Lectotype (male): NHMUK.

*Griselda antrifera* (Meyrick): Kuznetzov, 1979: 83.

*Rhopobota antrifera* (Meyrick): Brown, 1983: 100.

**Material examined.** 1 ♀, Hsinchu/Miaoli Kuangwu, 2000 m, 24-25-VI-1985, J. Heppner & H. Wang. [MGCL]. (Adult Number: TORT12182 ♀, Genitalia Slide Number: Gen. Sl. Bae UF–081 ♀)

**Distribution.** China (Fujian, Guangxi, Guizhou, Hubei, Taiwan, Zhejiang) and Russia.

**Remarks.** This species is recorded for the first time in Taiwan.

#### 3.21.2. *Rhopobota bicolor* Kawabe, 1989

*Rhopobota bicolor* Kawabe, 1989: 62. TL: Thailand, Chiang Mai, Doi Inthanon. Holotype (male): OPU.

**Material examined.** Paratypes: 1 ♂, Nantou Hsien, Lushan spa, 1200 m, 3-5-VIII-1985, A. Kawabe; 1 ♀, Bushi, Iruma, Saitama-ken, 1-VI-1979, H. Inoue. [USNM].

2 ♂♂, Nantou Hsien, Tsingjing Farm, 1900 m, 9-18-VI-1980, K. Fujisawa; 1 ♂, Nantou Hsien, Lushan spa, 1200 m, 7-8-VI-1988, K. Fujisawa; 1 ♀, Taipei Co., Chihsingshan, ca. 10 air km N Taipei, 700 m, 28-VII-1980, D. R. Davis; 1 ♀, Taipei Co., Chihsingshan, ca. 10 air km N Taipei, 700 m, 28-VII-1980, D. R. Davis. [USNM].

1 ♂, 2 ♀♀, Taipei Co., Wulai, 200 m, 17-19-VI-1985, J. Heppner & H. Wang; 1 ♀, Nantou Co., 5 km N Chitou, 500 m, 29-VI-1985, J. Heppner & H. Wang; 1 ♂, Nantou Co., Meifeng, 2100 m, 6-9-IX-1988, J. Heppner. [MGCL].

**Distribution.** China (Guizhou, Hubei, Hunan, Sichuan, Taiwan), Japan and Thailand.

#### 3.21.3. *Rhopobota naevana* (Hübner, [1814,1815,1816,1817])

*Tortrix unipunctana* Haworth, 1811[1812]: 454. TL: United Kingdom, Great Britain. Syntype(s) (unknown sex): NHMUK.

*Tortrix naevana* Hübner, [1814,1815,1816,1817]: pl. 41, figure 261. TL: Europe, Syntype(s) (unknown sex): Unknown.

*Lithographia geminana* Stephens, 1852: 99. TL: United Kingdom. Great Britain (Whittlesea, near Yorkshire). Syntype(s) (unknown sex): NHMUK.

*Rhopobota naevana* (Hübner): Lederer, 1859: 367.

*Sciaphila luctiferana* Walker, 1863: 342. TL: Canada. Hudson Bay, Albany River, St. Martin’s Falls. Holotype (female): NHMUK.

*Anchylopera vacciniana* Packard, 1869: 338. TL: USA. Massachusetts. Holotype (unknow sex): MCZ.

*Epinotia ilicifoliana* Kearfott, 1907: 158. TL: USA. North Carolina, Black Mountains, Mt Graybeard. Lectotype (male): AMNH.

*Laspeyresia malivorella* Matsumura, 1931: 1072. TL: Korea. Lectotype (male): EIHU.

*Acroclita microrrhyncha* Meyrick, 1931: 127. TL: India. Parachinar. Holotype (female): NHMUK.

*Rhopobota unipunctana* (Haworth): Brown, 1979: 21.

**Material examined.** 2 ♂♂, 3 ♀♀, Nantou Hsien, Lushan spa, 1200 m, 27-29-VII-1983, A. Kawabe; 1 ♀, Nantou Hsien, Lushan spa, 1200 m, 9-10-VIII-1987, A. Kawabe; 2 ♂♂, Nantou Hsien, Lushan spa, 1200 m, 7-8-VI-1988, K. Fujisawa; 1 ♂, Taipei Hsien, Wulai, 400 m, 14-VIII-1985, A. Kawabe; 1 ♀, Nantou Hsien, Tsingjing Farm, 1900 m, 9-VI-1988, K. Fujisawa; 1 ♂, 1 ♀, Nantou Co., Mei-feng, 30 km S Tayuling, 2200 m, 1-8-VI-1980, D. R. Davis; 1 ♂, Taiwan, S. Issiki; 1 ♀, Taiwan: Taihoku, 8-V-1946, S. Issiki. [USNM].

8 ♂♂, 6 ♀♀, Taipei Co., Wulai, 200 m, 17-19-VI-1985, J. Heppner & H. Wang; 1 ♂, Hsinchu Co., Kuangwu For. Sta., 2000 m, 18-25-VIII-1988, J. Heppner & H. Wang; 1 ♂, Taoyuan Co., Upper Palin, 1500 m, 7-9-VII-1985, J. Heppner & H. Wang; 1 ♀, Ilan Co., Tuchan, 480 m, 1-2-VII-1982, J. Heppner; 1 ♀, Taitung Co., Bishan Hot Spgs., 720 m, 26-29-X-1984, J. Heppner & H. Wang; 2 ♀♀, Hsinchu/Miaoli Kuangwu, 2000 m, 24-25-VI-1985, J. Heppner & H. Wang; 1 ♂, 1 ♀, Nantou Co., Lien-Hua-Chih For. Sta. Puli, 700 m, 4-VII-1996, J. Heppner & H. Wang. [MGCL].

**Host plants.** Oleaceae: *Fraxinus rhynchophylla* Hance, *F. mandshurica* Rupr.; Ericaceae: *Vaccinium vitis-idaea* Linn.; Rosaceae: *Malus mandshurica* (Maxim.) Kom., *M. spectabilis* (Ait.) Borkh., *Pyrus ussuriensis* Maxim., *Crataegus pinnatifida* Bunge, *Armeniaca vulgaris* Lam., *Prunus mume* Sieb. *et* Zucc., *Sorbus* sp.; Rhamnaceae: *Rhamnus davurica* Pall., *Ilex crenata* Thunb.; Oleaceae: *Syringa amurensis* Rupr. This species, known as the black-headed fireworm, is an important pest of apples, pears, and apricots.

**Distribution.** China (Anhui, Fujian, Gansu, Guangdong, Guizhou, Hebei, Heilongjiang, Henan, Hubei, Hunan, Inner Mongolia, Jiangxi, Jilin, Liaoning, Shaanxi, Sichuan, Taiwan, Tianjin, Xizang, Yunnan, Zhejiang), Europe, North America, India, Japan, Korea, Mongolia, Russia and Sri Lanka.

#### 3.21.4. *Rhopobota toshimai* (Kawabe, 1978)

*Griselda toshimai* Kawabe, 1978: 186. TL: Japan, Shikoku, Kagawa Prefecture, Okushoiri. Holotype (male): USNM.

*Epinotia toshimai* (Kawabe): Kawabe, 1992: 107.

*Rhopobota toshimai* (Kawabe): Brown, 1983: 104.

**Material examined.** 8 ♂♂, 1 ♀, Nantou Co., Mei-feng, 30 km S Tayuling, 2200 m, 1-8-VI-1980, D. R. Davis; 2 ♂♂, 1 ♀, Nantou Hsien, Tsuifeng, 2400 m, 29-XII-1989, A. Kawabe; 1 ♀, Nantou Hsien, Wushe, 1150 m, 27-XII-1988, A. Kawabe; 1 ♀, Nantou Hsien, Yushih, 1700 m, 3-4-VIII-1987, A. Kawabe; 1 ♂, Kansirei, 19-X-1934, S. Issiki; 1 ♂, Rantaisan, 16-V-1933, S. Issiki. [USNM].

14 ♂♂, 3 ♀♀, Nantou Co., Meifeng, 2100 m, 6-9-IX-1988, J. Heppner; 1 ♂, Hsinchu Co., Litonshan, 1450–1914 m, 13-VII-2005, J. Heppner; 1 ♂, Taichung Co., Chingshan, 1100 m, 31-VIII/4-IX-1988, J. Heppner & H. Wang; 1 ♀, Taichung Co., Lishan, 1800 m, 8-9-VII-2005, J. Heppner. [MGCL].

**Distribution.** China (Taiwan) and Japan.

### 3.22. Rhyacionia *Hübner, [1825]1816*

*Rhyacionia* Hübner, [1825]1816: 379. Type species: *Tortrix buoliana* Denis *et* Schiffermüller, 1775.

#### *Rhyacionia dativa* Heinrich, 1928

*Evertia japoniella* Matsumura, 1917: 516. TL: Japan. Holotype (unknown sex): Unknown.

*Rhyacionia dativa* Heinrich, 1928: 61. TL: Japan, Honshu, Kanagawa Prefecture, Yokohama. Holotype (male): USNM.

**Material examined.** 1 ♂, Hualien Hsien, Tayuling, 2560 m, 12-VI-1988, K. Fujisawa; 1 ♀, Hualien Hsien, Kuanyuan, 2400 m, 7-8-VIII-1987, A. Kawabe; 2 ♂♂, Nantou Hsien, Tsingjing Farm, 1900 m, 9-VI-1988, K. Fujisawa; 2 ♂♂, 1 ♀, Nantou Co., Tayuling, 2500 m, 9-18-VI-1980, D. R. Davis; 1 ♂, Taiwan: Baibara, 24-III-43, S. Issiki. [USNM].

1 ♂, Kaosiung Co., Liukuei, Shanpin For., 750 m, 16-23-III-1990, J. Heppner & H. Wang; 2 ♂♂, Nantou Co., Piluchi For. Sta. rd., 2500 m, 8-9-VII-2005, J. Heppner; 4 ♂♂, Hualien Co., Kuanyuan, Taroko N.P., 2375 m, 3-VII-1996, J. Heppner & H. Wang; 2 ♂♂, 8 ♀♀, Hualien Co., Kuanyuan, Taroko Gorge, 750 m, 6-7-VII-2005, J. Heppner; 1 ♂, Taichung Co., Chingshan, 1100 m, 8-11-V-1989, J. Heppner & H. Wang. [MGCL].

**Host plants.** Pinaceae: *Pinus massoniana* Lamb., *P. thunbergii* Parl.

**Distribution.** China (Anhui, Guangdong, Henan, Hubei, Jiangsu, Jiangxi, Jilin, Shandong, Sichuan, Taiwan, hejiang), Japan, Korea, Russia and Europe.

### 3.23. Spilonota *Stephens, 1829*

*Spilonota* Stephens, 1829: 173. Type species: *Tortrix ocellana* Denis *et* Schiffermüller, 1775.

*Tmetocera* Lederer, 1859: 124, 367. (Type species: [*Tortrix*] *comitana* Hübner, [1799] = *Tortrix ocellana* Denis *et* Schiffermüller, 1775).

#### 3.23.1. *Spilonota albicana* (Motschulsky, 1866)

*Grapholitha albicana* Motschulsky, 1866: 199. TL: Japan. Syntype(s) (unknown sex): Unknown.

*Grapholitha Tmetocera prognathana* Snellen, 1883: 227.

*Spilonota prognathana* Snellen, 1883: 227.

*Spilonota albicana* (Motschulsky): Kuznetzov, 1976: 78.

**Material examined.** 1 ♀, Nantou Hsien, Wushe, 1150 m, 1-V-90, A. Kawabe. [USNM]. (Adult Number: Sun-337 ♀)

1 ♂, Taichung Co., Chingshan, 1100 m, 8-11-V-1989, J. Heppner & H. Wang. [MGCL]. (Genitalia Slide Number: Gen. Sl. Bae UF-117 ♂)

**Host plants.** Rosaceae: *Malus pumila* Mill., *M. mandshurica* (Maxim.) Kom., *M. sieboldii* (Regel) Rehd., *M. pallasiana* Juz., *Pyrus* sp., *Photinia glabra* (Thunb.) Maxim., *Armeniaca vulgaris* Lamb., *Amygdalus persica* Linn., *Prunus salicina* Lindl., *P. serrulata* var. *spontanea* (Maxim.), *Cerasus pseudocerasus* (Lindl.) G. Don, *C. tomentosa* (Thunb.) Wall., *Cotoneaster melanocarpus* Lodd., *C. maximowiczii* Schneid., *C. dahurica* Koehne *et* Schneid., *Sorbus amurensis* Koehne, *Crataegus pinnatifida* Bunge; Pinaceae: *Larix gmelini* (Rupr.) Rupr., *Larix leptolepis* (Sieb. *et* Zucc.) Gord.; Betulaceae: *Corylus heterophylla* Fisch. *et* Bess.

**Distribution.** China (Fujian, Gansu, Guizhou, Hebei, Heilongjiang, Henan, Hubei, Hunan, Shaanxi, Sichuan, Taiwan, Tianjin, Zhejiang), Japan, Korea and Russia.

**Remarks.** This species is recorded for the first time in Taiwan.

#### 3.23.2. *Spilonota lechriaspis* Meyrick, 1932

*Spilonota lechriaspis* Meyrick, 1932: 306. TL: China, South Manchuria, Kwantung Mountains. Lectotype (male): NHMUK.

**Material examined.** 1 ♀, Nantou Hsien, Lushan, 1200 m, 7-8.VI-1988, K. Fujisawa. [USNM].

2 ♂♂, Kaohsiung Co., Liukuei, Shanpin For., 750 m, 16-23-III-1990, J. Heppner & H. Wang; 1 ♀, Nantou Hsien, Lushan spa, 1200 m, June 7-8, 1988, K. Fujisawa. [MGCL]. (Adult Number: TORT12092 ♂, Genitalia Slide Number: YH-Sun FL230 ♂)

**Host plants.** Rosaceae: *Malus pumila* Mill., *Eriobotrya japonica* (Thunb.) Lindl., *Crataegus pinnatifida* Bunge; Leguminosae: *Pyracantha angustifolia* (Franch.) Schneid.

**Distribution.** China (Fujian, Hebei, Heilongjiang, Henan, Shaanxi, Taiwan, Tianjin), Japan, Korea and Russia.

**Remarks.** This species is recorded for the first time in Taiwan.

#### 3.23.3. *Spilonota melanocopa* (Meyrick, 1912)

*Enarmonia melanocopa* Meyrick, 1912: 853. TL: India, Assam, Khasi Hills. Lectotype (male): NHMUK.

*Spilonota meleanocopa* Meyrick, 1912: 853.

**Material examined.** 1 ♂, Nantou Hsien, Nanshanchi, 25-26-VII-1983, A. Kawabe. [USNM].

2 ♀♀, Taichung Co., Chingshan, 1100 m, 31-VIII/4-IX-1988, J. Heppner & H. Wang. [MGCL].

**Distribution.** China (Taiwan) and India.

### 3.24. Strepsicrates *Meyrick, 1888*

*Monilia* Walker, 1866: 1741. Type species: *Monilia semicanella* Walker, 1866 [=*Strepsicrates smithiana* Walsingham, 1892].

*Strepsiceros* Meyrick, 1881: 678. Type species: *Sciaphila ejectana* Walker, 1863. [preoccupied]

*Strepsicrates* Meyrick, 1888: 73. [replacement name for *Strepsiceros*].

*Phthinolophus* Dyar, 1903: 207. Type species: *Phthinolophus indentanus* Dyar, 1903.

*Phthenolophus* Busck, 1910: 132. [misspelling of *Phthinolophus*].

*Sterpsiceros* Razowski, 1977: 285. [misspelling of *Strepsiceros*].

#### *Strepsicrates rhothia* (Meyrick, 1910)

*Spilonota rhothia* Meyrick, 1910: 368. TL: Ceylon [Sri Lanka], Maskeliya. Lectotype (male): NHMUK.

*Strepsicrates rhothia* (Meyrick): Clarke, 1958: 596.

**Material examined.** 1 ♂, Taihoku, 3-VII-1948, S. Issiki; 1 ♀, Taihoku, 30-V-1946, S. Issiki; 1 ♀, Taihoku, 8-IX-1946, S. Issiki; 1 ♂, Sirin, 18-IX-1933, S. Issiki. [USNM].

**Host plants.** Anacardiaceae: *Mangifera indica* L.; Coriariaceae: *Coriaria nepalensis* Wall.; Lythraceae: *Woodfordia fruticosa* (L.) Kurz.; Myrtaceae: *Eucalyptus alba* Reinw. ex Blume, *Eucalyptus citriodora* (Hook.) K.D. Hill & L.A.S. Johnson, *Melaleuca cajuputi* Powell, *Psidium guajava* L., *Syzygium cumini* (L.) Skeels, *Lophostemon confertus* (R.Br.) Peter G. Wilson & J.T. Waterh.

**Distribution.** China (Taiwan), Sri Lanka, India, the Democratic Republic of Congo, Ghana, Madagascar, Mauritius and South Africa.

### 3.25. Tritopterna *Meyrick, 1921*

*Tritopterna* Meyrick, 1921: 151.

Type species: *Tritopterna chionostoma* Meyrick, 1921.

#### *Tritopterna anachastopa* (Meyrick, 1934)

*Acroclita anachastopa* Meyrick, 1934: 483. TL: Indonesia, Java, Telawa. Lectotype (male): NHMUK.

*Tritopterna anachastopa* (Meyrick): Brown, 2005: 599.

**Material examined.** 1 ♂, 1 ♀, Hualien Hsien, Kuanyuan, 2400 m, 7-8-VIII-1987, A. Kawabe; 1 ♂, 1 ♀, Nantou Hsien, Lushan spa, 1200 m, 3-5-VIII-1985, A. Kawabe; 1 ♂, Nantou Hsien, Wushe, 1150 m, 27-XII-1988, A. Kawabe; 1 ♀, Hualien Hsien, Tayuling, 2560 m, 12-VI-1988, K. Fujisawa. [USNM].

**Host plants.** Euphorbiaceae: *Glochidion* sp., *Mallotus* sp.

**Distribution.** China (Taiwan) and Indonesia.

### 3.26. Zeiraphera *Treitschke, 1829*

*Zeiraphera* Treitschke, 1829: 231.

*Sinusia* Caradja, 1916: 61. Type species: *Steganoptycha imprimata* Caradja, 1916 [=*Grapholita Paedisca subcorticana* Snellen, 1883] [described as subgenus of *Steganoptycha*].

*Charlotta* Forbes, 1923: 379. Type species: *Phalaena Tortrix Coccyx ratzeburgiana* Saxesen *sensu* Forbes, 1923 [=*Zeiraphera canadensis* Mutuura *et* Freeman, 1966].

Type species: *Tortrix corticana* Denis *et* Schiffermüller *sensu* Hübner, [1812,1813] [=*Pyralis isertana* Fabricius, 1794].

#### 3.26.1. *Zeiraphera fulvomixtana* Kawabe, 1974

*Zeiraphera fulvomixtana* Kawabe, 1974: 98. TL: Japan, Honshu, Gunma Prefecture, Doaiguchi. Holotype (male): USNM.

**Material examined.** 2 ♀♀, Chiai Hsien, Fenchihu, 1400 m, 11-12-VIII-1985, A. Kawabe; 1 ♂, Nantou Hsien, Lushan spa, 1200 m, 3-5-VIII-1985, A. Kawabe; 11 ♂♂, 10 ♀♀, Chiai Hsien, Alishan, 2270 m, 8-10-VIII-1985, A. Kawabe; 2 ♂♂, 1 ♀, (Alishan) Chiai Hsien, Formosa, 12-VIII-1974, Y. Kishida; 3 ♀♀, (Lishan) Taichung Hsien, Formosa, 9-VIII-1974, Y. Kishida; 1 ♂, Alishan, Chiayi, Formosa, 11-VIII-1974, H. Nakajima; 2 ♂♂, Wulai, Taipei Hsien, Formosa, 1-VIII-1974, H. Nakajima; 4 ♂♂, Nantou Co., Tayuling, 2500 m, 9-18-VI-1980, D. R. Davis; 2 ♂♂, Nantou Hsien, Tsingjing Farm, 1900 m, 9-VI-1988, K. Fujisawa. [USNM].

1 ♂, Taichung Co., Chingshan, 1100 m, 31-VIII/4-IX-1988, J. Heppner & H. Wang; 1 ♂, 1 ♀, Taichung Co., Chingshan, 1100 m, 31-VIII/4-IX-1988, J. Heppner & H. Wang; 1 ♂, Nantou Co., Meifeng Hort. Sta., 2100 m, 6-10-IX-1988, J. Heppner & H. Wang; 1 ♂, Hsinchu Co., Kuangwu For. Sta., 2000 m, 18-25-VIII-1988, J. Heppner & H. Wang. [MGCL].

**Host plants.** Fagaceae: *Quercus glauca* Thunb.

**Distribution.** China (Taiwan), Japan and Korea.

#### 3.26.2. *Zeiraphera taiwana* Kawabe, 1986

*Zeiraphera taiwana* Kawabe, 1986: 79. TL: Taiwan, Chiai Hsien, Alishan. Holotype (male): USNM.

**Material examined.** Taiwan: holotype: ♂, Formosa [Taiwan], Alishan, Chiai Hsien, 12-VIII-1974, Y. Kishida. Paratype: 1 ♀, Formosa [Taiwan], Alishan, Chiayi, 2200 m, 9-11-VII-1964, H. Inoue. [USNM].

1 ♂, Kaohsiung Co., Tienchih, Yushan N.P., 2280 m, 7-VII-1996, J. Heppner & H. Wang; 2 ♂♂, 1 ♀, Hsinchu/Miaoli Kuangwu, 2000 m, 24-25-VI-1985, J. Heppner & H. Wang; 1 ♀, Hualien Co., 4 km E Tayuling, 2400 m, 5-IX-1988, J. Heppner & H. Wang. [MGCL].

**Distribution.** China (Taiwan).

#### 3.26.3. *Zeiraphera thymelopa* (Meyrick, 1938)

*Argyroploce thymelopa* Meyrick, 1938: 1. TL: China, Yunnan Province, Yi [Likiang]. Lectotype (male): NHMUK.

*Olethreutes thymelopa* (Meyrick): Clarke, 1958: 555.

*Zeiraphera hohuanshana* Kuznetzov, 1976: 21. TL: Taiwan, Hualien Hsien, Hohuanshan. Holotype: USNM, male.

*Zeiraphera hohuanshana* Kawabe, 1986: 79.

**Material examined.** Holotype: ♂, Hualien Hsien, Hohuanshan, 3100 m, 30-VII-1-VIII-1983, A. Kawabe. Paratypes: 1 ♂, 1 ♀, Hualien Hsien, Hohuanshan, 3100 m, 30-VII-1-VIII-1983, A. Kawabe. [USNM].

1 ♀, Hualien Hsien, Hohuanshan, 3100 m, 30-VII-1-VIII-1983 A. Kawabe; 2 ♂♂, Hualien Hsien, Kuanyuan, 2400 m, 7-8-VIII-1987, A. Kawabe. [USNM].

**Distribution.** China (Fujian, Gansu, Shaanxi, Taiwan, Xizang, Yunnan).

## 4. Conclusions

Despite its small size, Taiwan supports an exceptionally rich biodiversity with high levels of endemism. Of the approximately 4300 species of vascular plants, more than 1000 are endemic (https://lifeoftaiwan.com/about-taiwan/nature-ecology/, accessed on 30 July 2025). Approximately 30% of the insects on the island are found nowhere else, along with 20% of the mammals and 17% of the reptiles [15]. Of the approximately 500 species of butterflies recorded from Taiwan, 56 (11%) are unique to the island.

Four major terrestrial ecoregions are recognized on Taiwan [16]: (1) Jian Nan subtropical evergreen forests, (2) South China Sea Islands, (3) South Taiwan monsoon rain forests, and (4) Taiwan subtropical evergreen forests. From grassy lowlands to high-elevation mountains (reaching 3100 m elevation), countless plant communities and topographic diversity also contribute significantly to the overall species richness of the island.

This contribution, focused on the tortricid tribe Eucosmini, documents one species new to China and one genus and six species new to Taiwan Province. These discoveries continue to highlight the uniqueness and diversity of Taiwan’s insect fauna and illuminate Taiwan’s position in global patterns of biogeography. The detailed information presented herein represents the basic data for subsequent systematic and distributional research and will not only improve the taxonomic knowledge of the insects of China but will also contribute to the systematics and biogeography of Tortricidae globally.

In the present study, species identifications were based primarily on morphological characteristics. However, some specimens proved difficult to distinguish solely on the basis of morphology. As such, the taxonomic status of several problematic taxa will be further evaluated using molecular techniques in future research. Upcoming efforts will also focus on surveying remote and underexplored regions of Taiwan, which hold considerable potential for the discovery of new species and additional distribution records. By integrating molecular biology, ecological data, and multidisciplinary approaches, future studies can further elucidate the genetic diversity, adaptive mechanisms, and speciation processes within the tribe Eucosmini. These insights will provide a more robust foundation for both biodiversity conservation and the development of effective management strategies for economically relevant tortricid species.

## Data Availability

The raw data supporting the conclusions of this article will be made available by the authors on request.
